# Supplementary material for: MYC drives aggressive prostate cancer by disrupting transcriptional pause release at androgen receptor targets
Source: Nat Commun. 2022 May 13;13:2559. doi: 10.1038/s41467-022-30257-z (PMC9106722; doi:10.1038/s41467-022-30257-z)
Supplement: Supplementary file 1 — Supplementary Information [file 41467_2022_30257_MOESM1_ESM.pdf]

Supplementary Information

MYC drives aggressive prostate cancer by disrupting transcriptional pause release at androgen receptor targets

Xintao Qiu[1,2,†], Nadia Boufaied[3,†], Tarek Hallal[3,4], Avery Feit[1,2], Anna de Polo[3,5], Adrienne M. Luoma[6], Walaa Alahmadi[3,7], Janie Larocque[3,7], Giorgia Zadra[8,9], Yingtian Xie[1,2], Shengqing Gu[1,2,10], Qin Tang[1,2,10], Yi Zhang[1,10], Sudeepa Syamala[1], Ji-Heui Seo[2], Connor Bell[2], Edward O'Connor[2], Yang Liu[11], Edward M. Schaeffer[12], R. Jeffrey Karnes[13], Sheila Weinmann[14], Elai Davicioni[11], Colm Morrissey[15], Paloma Cejas[1,2], Leigh Ellis[16,17,18], Massimo Loda[19], Kai W. Wucherpfennig[6], Mark M. Pomerantz[2], Daniel E. Spratt[20], Eva Corey[15], Matthew L. Freedman[1,2,21], X. Shirley Liu[1,10], Myles Brown[1,2], Henry W. Long[1,2,‡,*], David P. Labbé[3,4,5,7,‡,*]

[1]Center for Functional Cancer Epigenetics, Dana-Farber Cancer Institute, Boston, MA, USA
[2]Department of Medical Oncology, Dana-Farber Cancer Institute, Harvard Medical School, Boston, MA, USA
[3]Cancer Research Program, Research Institute of the McGill University Health Centre, Montréal, Québec, Canada
[4]Department of Anatomy and Cell Biology, McGill University, Montréal, Québec, Canada
[5]Division of Urology, Department of Surgery, McGill University, Montréal, Québec, Canada
[6]Department of Cancer Immunology and Virology, Dana-Farber Cancer Institute, Harvard Medical School, Boston, MA, USA
[7]Division of Experimental Medicine, Department of Medicine, McGill University, Montréal, Québec, Canada
[8]Departments of Oncologic Pathology and Pathology, Dana-Farber Cancer Institute and Brigham's Women Hospital, Boston, MA, USA
[9]Institute of Molecular Genetics, Research National Council, Pavia, Italy
[10]Department of Data Science, Dana-Farber Cancer Institute, Harvard T.H. Chan School of Public Health, Boston, MA, USA
[11]Decipher Biosciences, San Diego, CA, USA
[12]Department of Urology, Northwestern University, Chicago, IL, USA
[13]Department of Urology, Mayo Clinic, Rochester, MN, USA
[14]Center for Health Research, Kaiser Permanente Northwest, Portland, OR, USA
[15]Department of Urology, University of Washington, Seattle, WA, USA
[16]Division of Medical Oncology, Department of Medicine, Cedars-Sinai Medical Center, Los Angeles, CA, USA
[17]Cedars-Sinai Samuel Oschin Comprehensive Cancer Institute, Los Angeles, CA, USA
[18]Center for Bioinformatics and Functional Genomics, Department of Biomedical Sciences, Cedars-Sinai Medical Center, Los Angeles, CA 90048, USA
[19]Department of Pathology and Laboratory Medicine, Weil Cornell Medicine, New York Presbyterian-Weill Cornell Campus, New York, NY, USA
[20]Department of Radiation Oncology, University Hospitals Seidman Cancer Center, Case Western Reserve University School of Medicine, Cleveland, OH, USA
[21]The Eli and Edythe L. Broad Institute, Cambridge, MA, USA
[†]These authors contributed equally
[‡]These authors jointly supervised this work
[*]E-Mail: henry_long@dfci.harvard.edu (H.W.L.), david.labbe@mcgill.ca (D.P.L.)

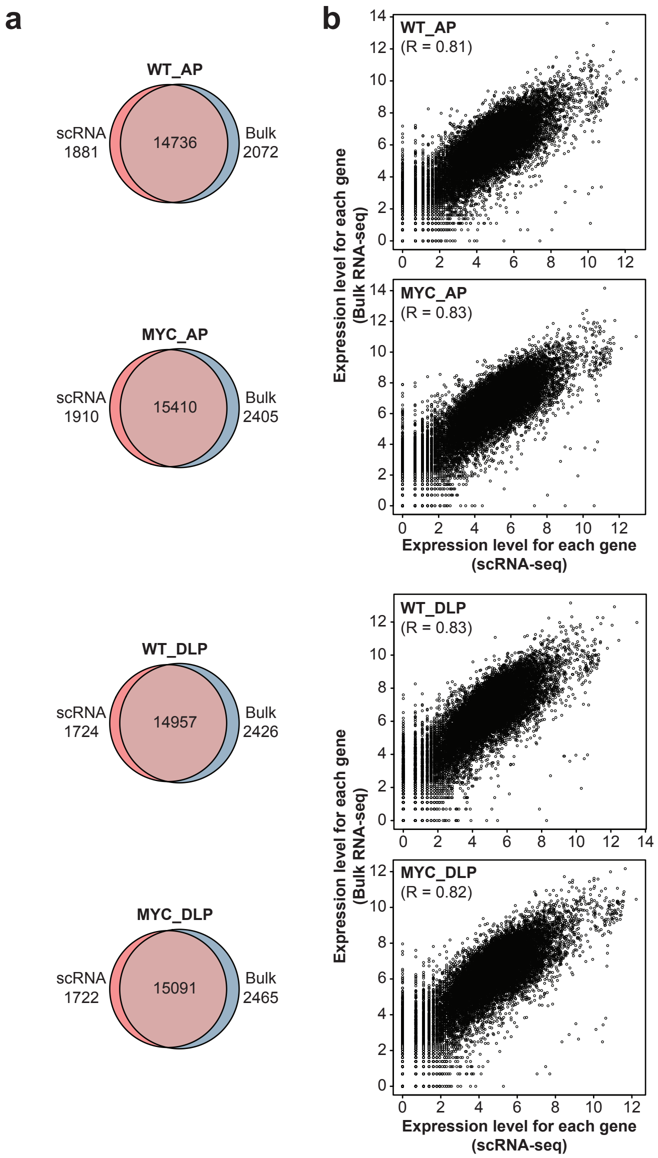

Supplementary Figure 1: Single-cell transcriptome is highly correlated with bulk gene expression in AP and DLP lobes. (**a, b**) Transcriptional profiling of WT and MYC-transformed AP and DLP lobes reveals high concordance for the total number of genes detected (**a**) and their expression levels (**b**) between bulk and single-cell RNA-seq (AP, DLP; matched bulk and single-cell RNA-seq; n = 1 per genotype). WT: wild-type; AP: anterior prostate; DLP: dorsolateral prostate.

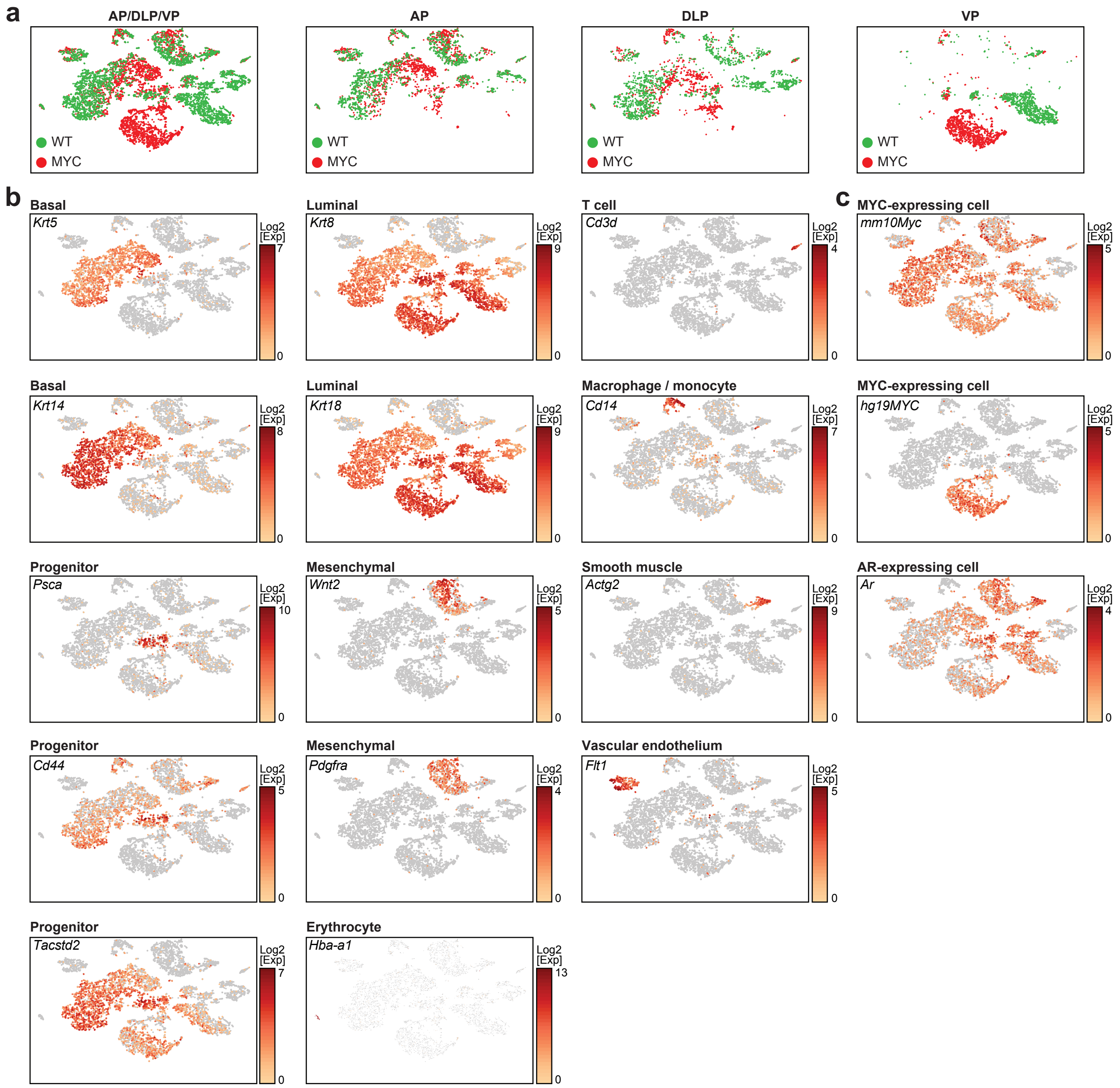

Supplementary Figure 2: Molecular characterization of murine WT and MYC-transformed prostate lobes. (**a**) Single-cell census of WT and MYC-transformed AP, DLP and VP. tSNE of scRNA-seq profiles (as in **Figure 2a**), colored by genotype (AP, DLP, VP; *n* = 1 per genotype). (**b, c**) Expression of selected markers of different subsets (**b**; AP, DLP, VP; *n* = 1 per genotype) as well as murine *Myc* (*mm10Myc*), human *MYC* (*hg19MYC*) and the *Ar* (**c**; AP, DLP, VP; *n* = 1 per genotype). WT: wild-type; VP: ventral prostate; DLP: dorsolateral prostate; AP: anterior prostate.

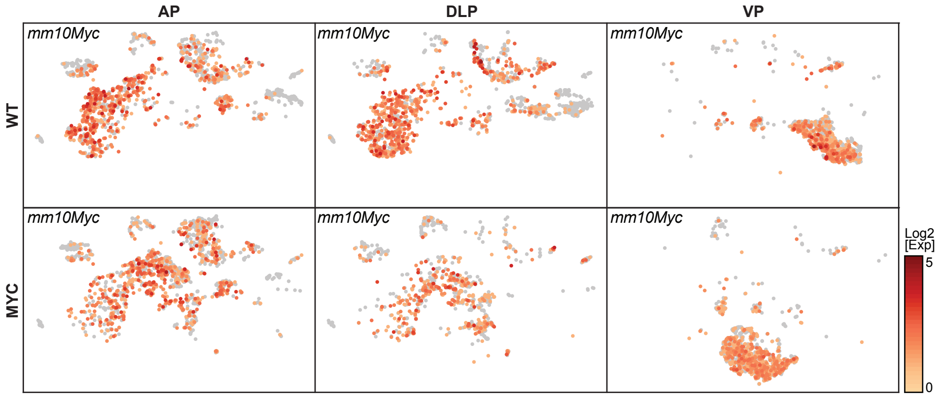

Supplementary Figure 3: Murine Myc is expressed across cell subpopulations and prostate lobes. Expression of murine *Myc* (*mm10Myc*) in WT and MYC-transformed AP, DLP and VP (AP, DLP, VP; *n* = 1 per genotype). WT: wild-type; AP: anterior prostate; DLP: dorsolateral prostate; VP: ventral prostate.

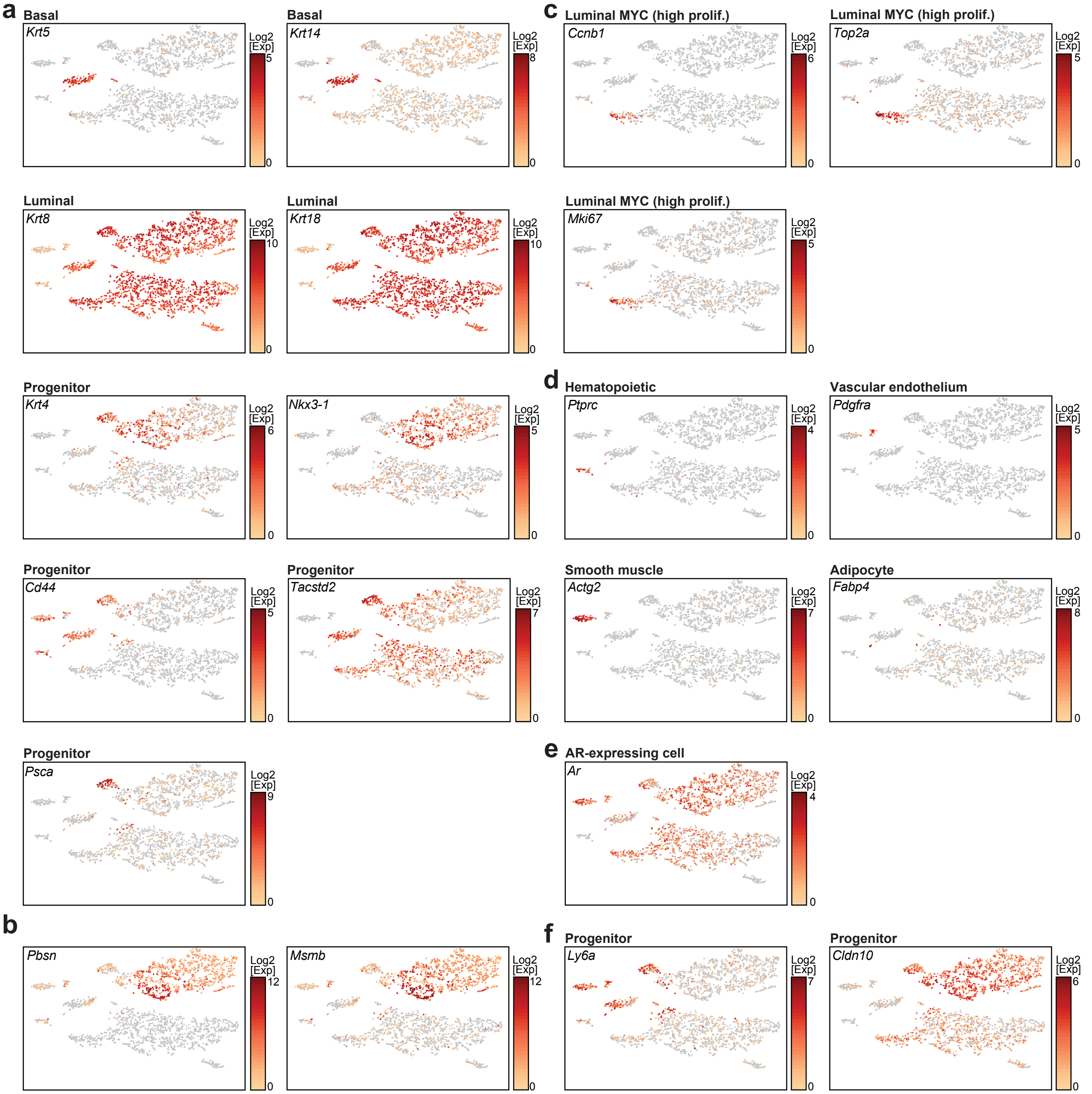

Supplementary Figure 4: Molecular characterization of murine WT and MYC-transformed VP. (**a-f**) Expression of selected markers of different subsets (VP; *n* = 1 per genotype). WT: wild-type; VP: ventral prostate.

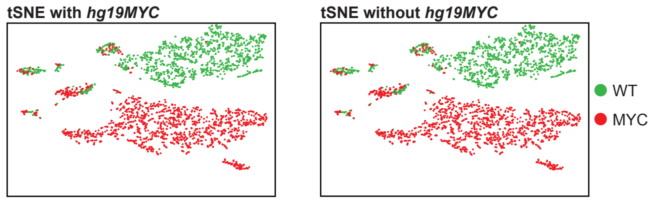

Supplementary Figure 5: tSNE of scRNA-seq profiles is not affected by the inclusion of human *MYC* transcript. tSNE of VP generated with (*left*) or without (*right*) the inclusion of human *MYC* (*hg19MYC*; VP; *n* = 1 per genotype). WT: wild-type; VP: ventral prostate.

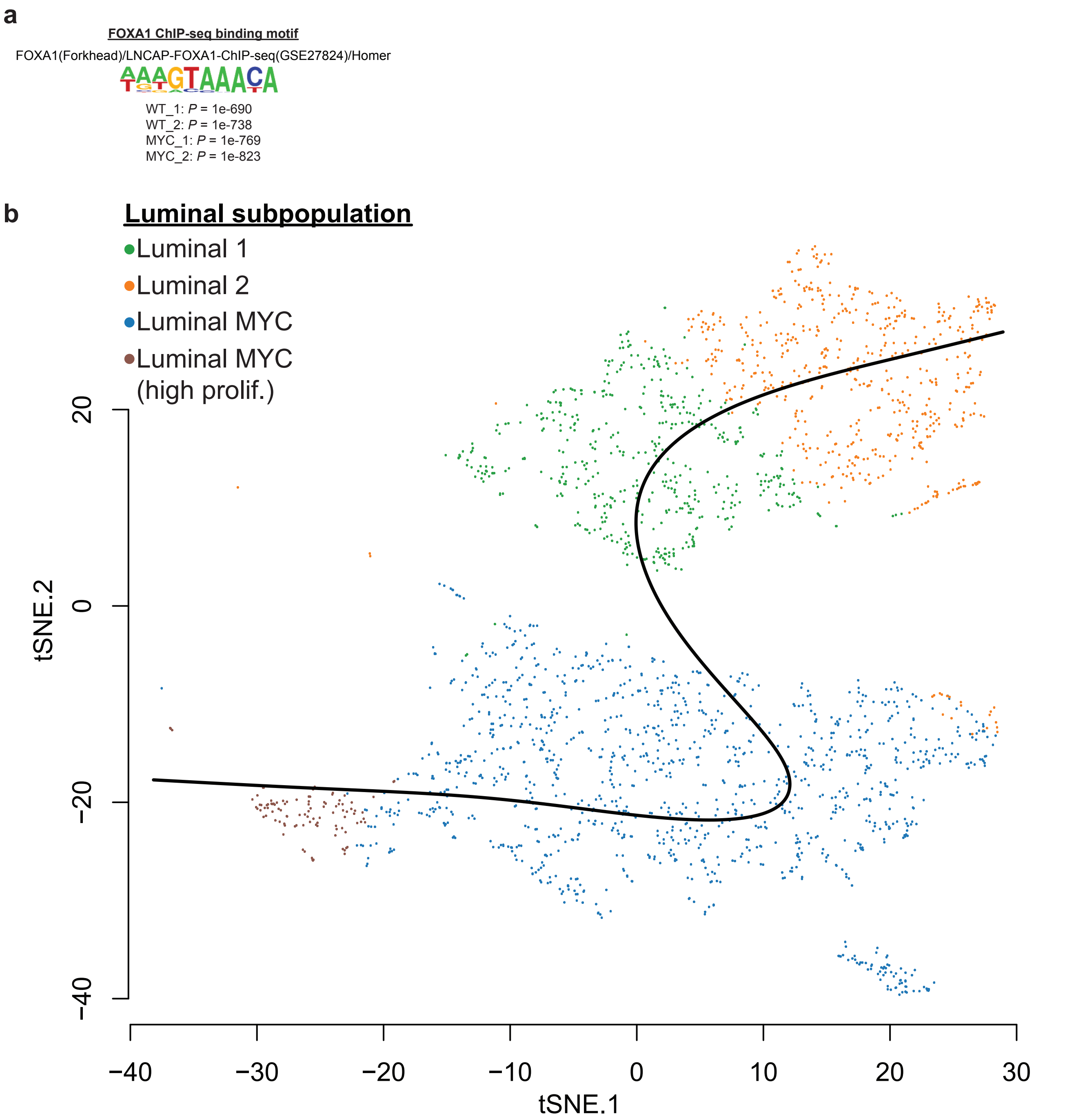

Supplementary Figure 6: Integration of ChIP-seq with scRNA-seq. (**a**) FOXA1 ChIP-seq identifies FHRE as the top FOXA1 binding motif in WT and MYC-transformed VP (VP; *n* = 2 pools of biological replicates (*n* = 8-13) per genotype). (**b**) Slingshot pseudotime inference used to order luminal cells in **Figure 4g** (VP; *n* = 1 per genotype). WT: wild-type; VP: ventral prostate; FHRE: forkhead response element.

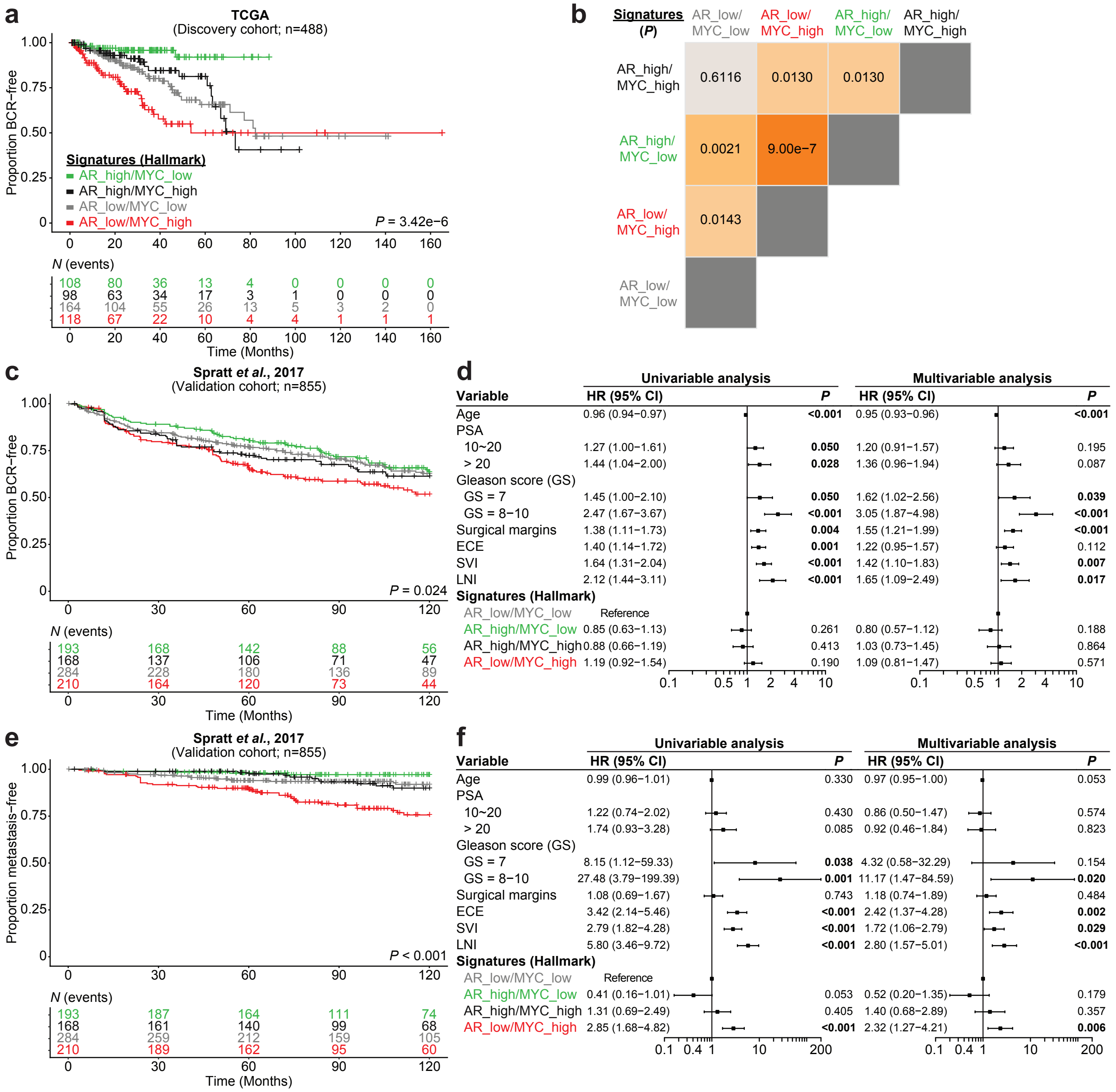

Supplementary Figure 7: Divergent MYC and AR transcriptional programs dictate disease progression. (**a, b**) Kaplan-Meier curves (**a**) and log-rank tests (**b**) reveal that patients bearing a primary tumor characterized by low AR transcriptional signature (Hallmark) and concurrent high MYC transcriptional signature (Hallmark) have a shorter time to biochemical recurrence (BCR) within the discovery cohort (TCGA). (**c, d**) Kaplan-Meier curves (**c**) but not univariable and multivariable analysis (**d**; Cox proportional hazards model) confirms that tumors with concurrent low AR and high MYC transcriptional signatures have a significant more rapid development of BCR than tumors with low AR transcriptional signature without an active MYC transcriptional program in the validation cohort (Spratt *et al.*, 2017 [1]; n = 855; HR ± 95% CI). (**e, f**) Kaplan-Meier curves (**e**), univariable and multivariable analyses (**f**; Cox proportional hazards model) reveal that tumors with concurrent low AR and high MYC transcriptional signatures are more likely to develop metastatic disease (n = 855; HR ± 95% CI). PSA: prostate-specific antigen; HR: hazard ratio; CI: confidence interval; GS: Gleason score; ECE: extracapsular extension; SVI: seminal vesicles invasion; LNI: lymph node involvement.

a

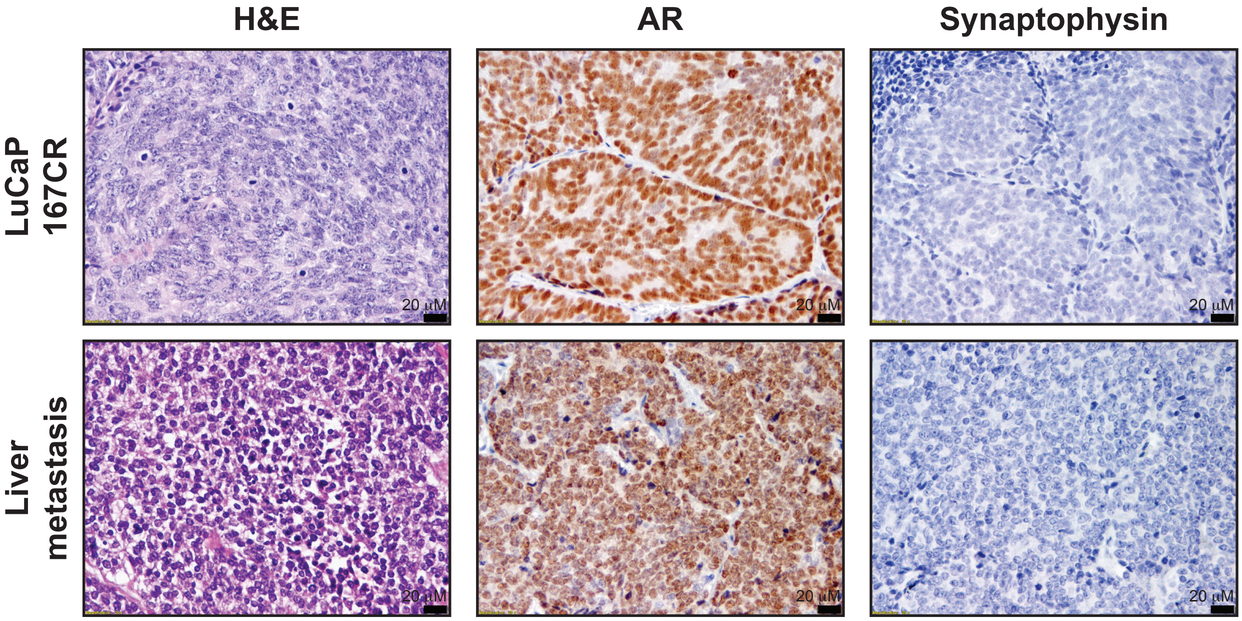

b

AR ChIP-seq binding motif

ARE(NR)/LNCAP-AR-ChIP-seq(GSE27824)/Homer

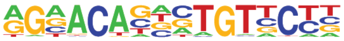

MYC-low		*MYC*-high	
PDX	*P*	PDX	*P*
105CR	1e-673	167CR	1e-1322
136CR	1e-628	70CR	1e-706
147CR	1e-318	78CR	1e-666
96CR	1e-327	81CR	1e-641

c

FOXA1 ChIP-seq binding motif

FOXA1(Forkhead)/LNCAP-FOXA1-ChIP-seq (GSE27824)/Homer

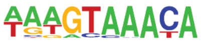

MYC-low		*MYC*-high	
PDX	*P*	PDX	*P*
105CR	1e-729	167CR	1e-693
136CR	1e-801	70CR	1e-869
147CR	1e-916	78CR	1e-558
96CR	1e-764	81CR	1e-658

Supplementary Figure 8: mCRPC LuCaP patient-derived xenograft (PDX) 167CR characterization and top binding motifs in LuCaP PDXs AR and FOXA1 ChIP-seq. (**a**) LuCaP PDX 167CR was established from a liver metastasis of a male who died of abiraterone-, carboplatin- and docetaxel-resistant CRPC. LuCaP 167CR expresses AR, responds to castration and is negative for synaptophysin. Morphology of the PDX was concordant with the original liver metastasis (**b, c**) AR and FOXA1 ChIP-seq identifies ARE (**b**) and FHRE (**c**), respectively as the top binding motif in mCRPC LuCaP PDXs. ARE: androgen response element; FHRE: forkhead response element.

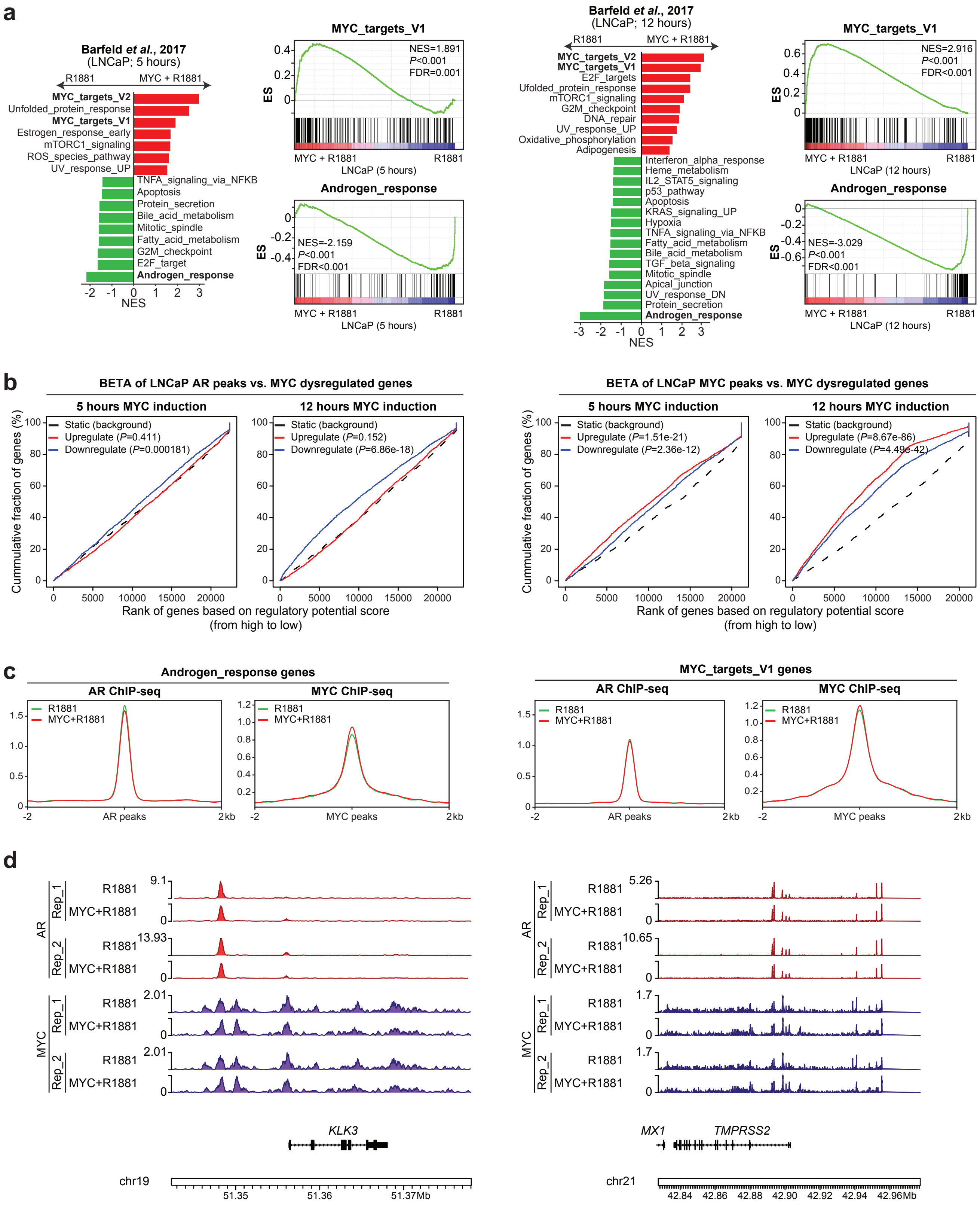

Supplementary Figure 9

Supplementary Figure 9: MYC overexpression disrupts the AR transcriptional program in LNCaP cells. Reanalysis of transcriptomics and epigenetics data from Barfeld and colleagues [2]. (**a**) Gene Set Enrichment Analysis (GSEA, Hallmark, P<0.05 and FDR<0.1) revealed an enriched MYC transcriptional program and a depleted AR response following 5 or 12 hours of MYC induction (P<0.001 and FDR<0.001; Source data are provided as a Source Data file). (**b**) BETA analysis revealed that AR binding sites are associated with gene downregulation while MYC binding sites are associated with gene upregulation following MYC induction. (**c**) AR and MYC binding nearby Androgen_response and MYC_targets_V1 genes is unchanged following MYC induction despite a dampened AR and a heightened MYC transcriptional program. (**d**) Unchanged AR and MYC binding at *KLK3* and *TMPRSS2* loci, AR-dependent genes downregulated by MYC overexpression. NES: normalized enrichment score; ES: enrichment score.

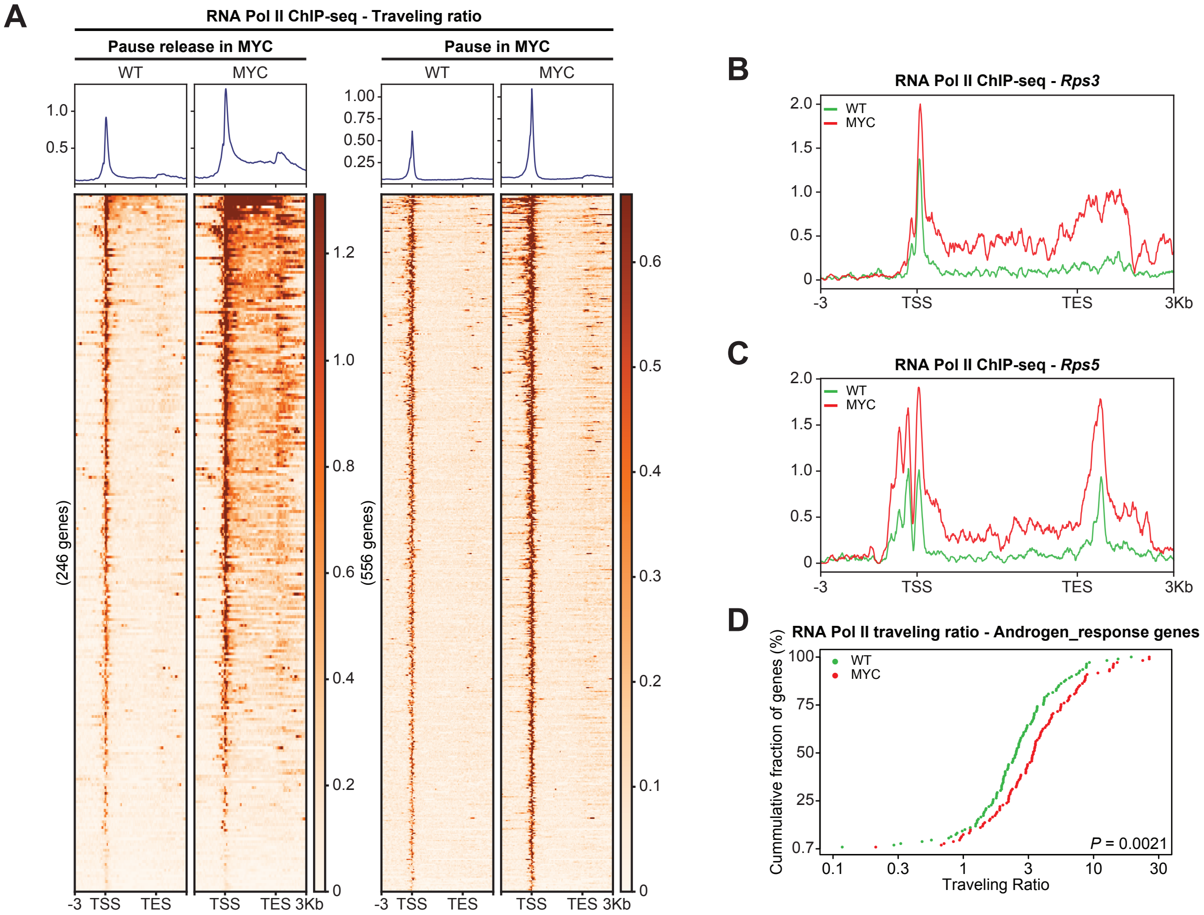

Supplementary Figure 10: RNA Pol II promoter-proximal pausing. (**a**) RNA Pol II occupancy at pause release (*left*) and pause genes (*right*) following MYC overexpression (VP; *n* = 2 pools of biological replicates (*n* = 8-13) per genotype). (**b, c**) Pause release at *Rps3* (**b**) and *Rps6* (**c**) MYC_targets_V1 genes (VP; *n* = 2 pools of biological replicates (*n* = 8-13) per genotype). (**d**) RNA Pol II traveling ratio reveals greater promoter-proximal pausing at Androgen_response genes (non-smoothed curves. TSS: transcription start site; TES: transcription end site.

SUPPLEMENTARY REFERENCES

1 Spratt, D. E. *et al.* Individual Patient-Level Meta-Analysis of the Performance of the Decipher Genomic Classifier in High-Risk Men After Prostatectomy to Predict Development of Metastatic Disease. *J Clin Oncol* **35**, 1991-1998, doi:10.1200/JCO.2016.70.2811 (2017).

2 Barfeld, S. J. *et al.* c-Myc Antagonises the Transcriptional Activity of the Androgen Receptor in Prostate Cancer Affecting Key Gene Networks. *EBioMedicine* **18**, 83-93, doi:10.1016/j.ebiom.2017.04.006 (2017).
