# Supplementary material for: MYC drives aggressive prostate cancer by disrupting transcriptional pause release at androgen receptor targets
Source: Nat Commun. 2022 May 13;13:2559. doi: 10.1038/s41467-022-30257-z (PMC9106722; doi:10.1038/s41467-022-30257-z)
Supplement: Supplementary file 3 — Reporting Summary [file 41467_2022_30257_MOESM3_ESM.pdf]

nature research

Corresponding author(s): Henry W. Long, David P. Labbé

Last updated by author(s): Apr 11, 2022

Reporting Summary

Nature Research wishes to improve the reproducibility of the work that we publish. This form provides structure for consistency and transparency in reporting. For further information on Nature Research policies, see our Editorial Policies and the Editorial Policy Checklist.

Statistics

For all statistical analyses, confirm that the following items are present in the figure legend, table legend, main text, or Methods section.

n/a	Confirmed	
☐	☒	The exact sample size (*n*) for each experimental group/condition, given as a discrete number and unit of measurement
☐	☒	A statement on whether measurements were taken from distinct samples or whether the same sample was measured repeatedly
☐	☒	The statistical test(s) used AND whether they are one- or two-sided<br>*Only common tests should be described solely by name; describe more complex techniques in the Methods section.*
☐	☒	A description of all covariates tested
☐	☒	A description of any assumptions or corrections, such as tests of normality and adjustment for multiple comparisons
☐	☒	A full description of the statistical parameters including central tendency (e.g. means) or other basic estimates (e.g. regression coefficient) AND variation (e.g. standard deviation) or associated estimates of uncertainty (e.g. confidence intervals)
☐	☒	For null hypothesis testing, the test statistic (e.g. *F*, *t*, *r*) with confidence intervals, effect sizes, degrees of freedom and *P* value noted<br>*Give P values as exact values whenever suitable.*
☒	☐	For Bayesian analysis, information on the choice of priors and Markov chain Monte Carlo settings
☒	☐	For hierarchical and complex designs, identification of the appropriate level for tests and full reporting of outcomes
☐	☒	Estimates of effect sizes (e.g. Cohen's *d*, Pearson's *r*), indicating how they were calculated

Our web collection on statistics for biologists contains articles on many of the points above.

Software and code

Policy information about availability of computer code

Data collection	Raw Illumina output was converted to fastq format using Illumina Bcl2fastq v2.18.
Data analysis	ChiLin v2.0 (http://cistrome.org/chilin/) for ChIP-seq QC; BWA v0.7.17-r1188 (http://bio-bwa.sourceforge.net/) for mapping; MACS2 v2.1.1.20160309 (http://liulab.dfci.harvard.edu/MACS/) for peak calling; DESeq2 v1.18.1 (https://bioconductor.org/packages/release/bioc/html/DESeq2.html) for differential peak analysis; HOMER v3.0.0 (http://homer.ucsd.edu/homer/motif/ -) for motif enrichment analysis; 10x Cell Ranger v2.0.0 (https://support.10xgenomics.com/single-cell-gene-expression/software/downloads/latest) for preprocessing scRNAdata; GSEA v4.1.0 (http://www.gsea-msigdb.org/gsea/downloads.jsp) for pathway analysis; Slingshot v1.3.1 (https://github.com/kstreet13/slingshot) for Pseudotime inference; VIPER v2.0 (https://bitbucket.org/cfce/viper/src/master/) for read alignment, quality control and data analysis; STAR v2.7.0f (https://github.com/alexdobin/STAR) for RNA-seq read mapping; Cufflinks v2.2.1 (http://cole-trapnell-lab.github.io/cufflinks/) for RNA-seq readcounts; SVA v.3.18.00 for scRNA-seq and RNA-seq batch effects removal; CoBRA v2.0 (https://cfce-cobra.readthedocs.io/) for ChIP-seq sample-sample correlation and differential peaks analysis; deepTools v2.3.5 (https://deeptools.readthedocs.io/) for ChIP-seq profiling; BETA v1.0.7 (http://cistrome.org/BETA/) integrate ChIP-seq and gene expression.<br><br>The following R packages were used: Seurat (v3.1.1) for scRNA-seq unsupervised clustering; ggplot2 (v3.3.2) for visualization; stats (v3.6.0) for covariance analysis; ComplexHeatmap (v2.2.0) for heatmap visualization; karyoploteR (v1.12.4) for ChIP-seq profile visualization; Bioconductor TCGAbiolinks (v2.14.1) for retrieving RNA-seq readcounts and clinical data from TCGA; singscore (v1.6.0) for generation of transcriptional signature scores; maxstat (v0.7-25) for defining cut-off point of transcriptional signature scores; survival (v3.2-3) for performing survival analysis; survminer (v0.4.8) for plotting Kaplan-Meier; scan.upc (v2.28.0) for normalizing microarray expression levels.

For manuscripts utilizing custom algorithms or software that are central to the research but not yet described in published literature, software must be made available to editors and reviewers. We strongly encourage code deposition in a community repository (e.g. GitHub). See the Nature Research guidelines for submitting code & software for further information.

Data

Policy information about availability of data

All manuscripts must include a data availability statement. This statement should provide the following information, where applicable:

- Accession codes, unique identifiers, or web links for publicly available datasets
- A list of figures that have associated raw data
- A description of any restrictions on data availability

The murine (bulk RNA-seq, scRNA-seq and ChIP-seq) and LuCaP PDXs (ChIP-seq) sequencing data reported in this paper were deposited on NCBI Gene Expression Omnibus (GEO) and are accessible through GEO Series accession number GSE163146 (https://www.ncbi.nlm.nih.gov/geo/query/acc.cgi?acc=GSE163146) and GSE163220 (https://www.ncbi.nlm.nih.gov/geo/query/acc.cgi?acc=GSE163220), respectively. The CRPC (bulk RNA-seq) and the LNCaP MYC model (microarray and ChIP-seq) publicly available data used in this study are available through GEO Series accession number GSE126078 (https://www.ncbi.nlm.nih.gov/geo/query/acc.cgi?acc=GSE126078) and GSE73995 (https://www.ncbi.nlm.nih.gov/geo/query/acc.cgi?acc=GSE73995), respectively. The remaining data are available within the Article, Supplementary Information or Source Data file provided with this paper.

Field-specific reporting

Please select the one below that is the best fit for your research. If you are not sure, read the appropriate sections before making your selection.

☒ Life sciences ☐ Behavioural & social sciences ☐ Ecological, evolutionary & environmental sciences

For a reference copy of the document with all sections, see nature.com/documents/nr-reporting-summary-flat.pdf

Life sciences study design

All studies must disclose on these points even when the disclosure is negative.

Sample size	Sample size for scRNA-seq was estimated empirically for sufficient statistical power based on literature. Sample size for RNA-seq and ChIP-seq was determined based on previous analyses (Labbé et al., Nature Communications, 2019) demonstrating ample sample sizes for identifying transcriptomic and cistromic changes.
Data exclusions	No data were excluded from the analysis.
Replication	VP, DPL and AP biospecimens for scRNA-seq were harvested from a single WT or Hi-MYC mouse. The impact of MYC overexpression on the Androgen_response was replicated using data from Barfeld et al., Labrecque et al. and the LuCaP PDXs series. Protein expression in VP was assessed from three biological independent biospecimens. The murine ChIP-seq analyses reported comparing genotype conditions uses two pools of biological replicates comprised of at least 8 samples each harvested from distinct mice and key results were replicated using data from Barfeld et al. and the LuCaP PDXs series. Number of independent samples is clearly stated in the manuscript.<br>The combination of the Hallmark Androgen_response / Hallmark MYC_targets_V1 and AR-A / Hallmark MYC_targets_V1 signatures and their association with prostate cancer progression was examined (and replicated) in the META855 cohort using the thresholds obtained from quantiles defined in the TCGA dataset (discovery cohort). This finding was also replicated in a mCRPC cohort (Abida et al.).
Randomization	Randomization was not relevant to this study because comparisons were across distinct conditions.
Blinding	Histopathological slides were analysed by expert murine uropathologist, who were blind to the experimental conditions. Investigators were blind to the results obtained from the discovery cohort (TCGA) for the validation analysis (META855 cohort). All other experiments were performed in a non-blind fashion in order to perform comparisons across distinct conditions or because of the laboratory structure making blinding impractical.

Reporting for specific materials, systems and methods

We require information from authors about some types of materials, experimental systems and methods used in many studies. Here, indicate whether each material, system or method listed is relevant to your study. If you are not sure if a list item applies to your research, read the appropriate section before selecting a response.

Materials & experimental systems

n/a	Involved in the study	
☐	☒	Antibodies
☒	☐	Eukaryotic cell lines
☒	☐	Palaeontology and archaeology
☐	☒	Animals and other organisms
☐	☒	Human research participants
☒	☐	Clinical data
☒	☐	Dual use research of concern

Methods

n/a	Involved in the study	
☐	☒	ChIP-seq
☒	☐	Flow cytometry
☒	☐	MRI-based neuroimaging

Antibodies

Antibodies used

We have used only commercial commercially available antibodies. List of antibodies used:
1) anti-AR: #ab108341, Abcam;
2) anti-AR: N-20; #sc-816, Santa Cruz Biotechnology;
3) anti-c-MYC: #ab32072, Abcam;
4) anti-beta-Actin: #4967, Cell Signaling Technology;
5) anti-FOXA1: #ab23738, Abcam;
6) anti-H3K27ac: #ab4729, Abcam;
7) anti-H3K27ac: #C15410196, Diagenode;
8) anti-RNA Pol II: #sc899, Santa Cruz Biotechnology;
9) anti-AR: #MU256-UC, Biogenex;
10) anti-synaptophysin: #sc-17750, Santa Cruz Biotechnology.

Validation

All the antibodies are widely published and have quality control tested per the company's standard procedure:
1) anti-AR: #ab108341 [ER179(2)], Abcam (reference - PMID 34417459; product datasheet - https://www.abcam.com/androgen-receptor-antibody-er1792-chip-grade-ab108341.html);
2) anti-AR: N-20; #sc-816, Santa Cruz Biotechnology (reference - PMID 26457646; product datasheet - https://www.scbt.com/scbt/product/ar-antibody-n-20);
3) anti-c-MYC: #ab32072 [Y69], Abcam (reference - PMID 31554818; product datasheet - https://www.abcam.com/c-myc-antibody-y69-ab32072.html);
4) anti-beta-Actin: #4967, Cell Signaling Technology (reference - PMID 31554818; product datasheet - https://www.cellsignal.com/products/primary-antibodies/b-actin-antibody/4967);
5) anti-FOXA1: #ab23738, Abcam (reference - PMID 26457646; product datasheet - https://www.abcam.com/foxa1-antibody-chip-grade-ab23738.pdf);
6) anti-H3K27ac: #ab4729, Abcam (reference - PMID 33750801 product datasheet - https://www.abcam.com/histone-h3-acetyl-k27-antibody-chip-grade-ab4729.html);
7) anti-H3K27ac: #C15410196, Diagenode (reference - PMID 30773341; product datasheet - https://www.diagenode.com/en/p/h3k27ac-polyclonal-antibody-premium-50-mg-18-ml#);
8) anti-RNA Pol II: #sc899, Santa Cruz Biotechnology (reference - PMID 20434984; product datasheet - https://datasheets.scbt.com/sc-899.pdf);
9) anti-AR: #MU256-UC [F39.4.1], Biogenex (reference - PMID 31361600; product datasheet - http://store.biogenex.com/us/applications/ihc/controls/controls/anti-androgen-receptor-clone-f39-4-1.html);
10) anti-synaptophysin: #sc-17750 [D-4], Santa Cruz Biotechnology (reference - PMID 31361600; product datasheet - https://www.scbt.com/p/syp-antibody-d-4).

Animals and other organisms

Policy information about studies involving animals; ARRIVE guidelines recommended for reporting animal research

Laboratory animals

FVB Hi-MYC (strain number 01XK8), expressing the human c-MYC transgene in prostatic epithelium (heterozygous for MYC transgene), were obtained from the National Cancer Institute Mouse Repository at Frederick National Laboratory for Cancer Research. Hi-MYC mice were bred with wild-type FVB mice to obtain male wild-type and Hi-MYC mice to be used for this research. At 12 weeks of age, male mice were euthanized and tissues were collected.

Wild animals

No wild animals were used in this study.

Field-collected samples

No field collected samples were used in the study.

Ethics oversight

The animal protocol (#13-049) was reviewed and approved by the Dana-Farber Cancer Institute Institutional Animal Care and Use Committee (IACUC), and was in accordance with the Animal Welfare Act. The animal protocol (#2017-7961) also followed the ethical guidelines of the Canadian Council on Animal Care, and was approved by the Research Institute of the McGill University Health Centre Glen Facility Animal Care Committee (FACC).

Note that full information on the approval of the study protocol must also be provided in the manuscript.

Human research participants

Policy information about studies involving human research participants

Population characteristics

Informed consent was obtained to collect human mCRPC tissues and generate the patient-derived xenograft tumors as described previously (Labrecque et al., J Clin Oncol, 2017; Nguyen et al., Prostate, 2017). The study was approved by the University of Washington Human Subjects Division Institutional Review Board (no. 2341). All patients were males with advanced prostate cancer that received androgen ablation therapy. Age at diagnosis was captured as well as the source of the tissue.

Recruitment

Patients were approached and enrolled in the prostate cancer donor program by their oncologist at the University of Washington. After confirming consent with the patient and/or the immediate family of a patient with advanced disease, tumor specimens were collected after death. While efforts to recruit minority patients are ongoing, the patient population at the University of Washington and the Seattle catchment area for the program are predominantly white. This leads to a selection bias towards white patients within the patient cohort.

Ethics oversight | University of Washington Human Subjects Division Institutional Review Board.

Note that full information on the approval of the study protocol must also be provided in the manuscript.

ChIP-seq

Data deposition

☒ Confirm that both raw and final processed data have been deposited in a public database such as GEO.

☒ Confirm that you have deposited or provided access to graph files (e.g. BED files) for the called peaks.

Data access links
May remain private before publication.

GSE163146; https://www.ncbi.nlm.nih.gov/geo/query/acc.cgi?&acc=GSE163146
GSE163220; https://www.ncbi.nlm.nih.gov/geo/query/acc.cgi?acc=GSE163220

Files in database submission

fastq and bigWig files for the following ChIP-Seq samples were deposited in the Gene Expression Omnibus, they can be accessed using the link above:
GSM4972719 FOXA1_WT_1
GSM4972720 FOXA1_WT_2
GSM4972721 FOXA1_MYC_1
GSM4972722 FOXA1_MYC_2
GSM4972723 AR_WT_1
GSM4972724 AR_WT_2
GSM4972725 AR_MYC_1
GSM4972726 AR_MYC_2
GSM4972727 H3K27ac_WT_1
GSM4972728 H3K27ac_WT_2
GSM4972729 H3K27ac_MYC_1
GSM4972730 H3K27ac_MYC_2
GSM4972731 Pol2_WT_1
GSM4972732 Pol2_WT_2
GSM4972733 Pol2_MYC_1
GSM4972734 Pol2_MYC_2
GSM4975226 AR_105CR
GSM4975227 AR_136CR
GSM4975228 AR_147CR
GSM4975229 AR_167CR
GSM4975230 AR_70CR
GSM4975231 AR_78CR
GSM4975232 AR_81CR
GSM4975233 AR_96CR
GSM4975234 FOX_105CR
GSM4975235 FOX_136CR
GSM4975236 FOX_147CR
GSM4975237 FOX_167CR
GSM4975238 FOX_70CR
GSM4975239 FOX_78CR
GSM4975240 FOX_81CR
GSM4975241 FOX_96CR
GSM4975242 K27ac_105CR
GSM4975243 K27ac_136CR
GSM4975244 K27ac_147CR
GSM4975245 K27ac_167CR
GSM4975246 K27ac_70CR
GSM4975247 K27ac_78CR
GSM4975248 K27ac_81CR
GSM4975249 K27ac_96CR

Genome browser session
(e.g. UCSC)

http://genome.ucsc.edu/cgi-bin/hgTracks?db=mm9&hubUrl=https://data.cyverse.org/dav-anon/iplant/home/dfcicancer/trackhub/hub.txt

Methodology

Replicates

Merged two pools of biological replicates each comprised of multiple samples harvested from distinct 12-week-old mice:
WT Pool #1 (12 mice) and Pool #2 (13 mice);
MYC Pool #3 (8 mice) and Pool #4 (8 mice).

Sequencing depth

All ChIP-Seq samples were single-end sequenced, the raw and mapped read number for each are below:
Sample Name Raw Reads Mapped Reads

AR_MYC_1 34269574 32910932
AR_MYC_2 85071572 81362447
AR_WT_1 58293707 55870686
AR_WT_2 52273701 50212995
FOXA1_MYC_1 45771664 45072170
FOXA1_MYC_2 50567597 49897725
FOXA1_WT_1 53339419 52656581
FOXA1_WT_2 53246498 52409357
H3K27ac_MYC_1 49957249 48541056
H3K27ac_MYC_2 61726803 60260267
H3K27ac_WT_1 54053305 52413951
H3K27ac_WT_2 50419559 49289837
Pol2_MYC_1 87184435 84359846
Pol2_MYC_2 72349486 69274722
Pol2_WT_1 69606185 68054299
Pol2_WT_2 64600151 62714578
AR_105CR 20012067 19143950
AR_136CR 20010423 18179250
AR_147CR 20015887 15954896
AR_167CR 20012805 17868349
AR_70CR 20010201 17458893
AR_78CR 20011251 17615152
AR_81CR 20012482 16627634
AR_96CR 20009516 19603244
FOX_105CR 20008295 16522341
FOX_136CR 20009033 17944989
FOX_147CR 20009040 16999179
FOX_167CR 20013152 18380360
FOX_70CR 20009210 17635278
FOX_78CR 20278702 16673554
FOX_81CR 20010827 18375494
FOX_96CR 20010311 16492714
K27ac_105CR 20008342 17477192
K27ac_136CR 20010902 18542759
K27ac_147CR 20009922 16245965
K27ac_167CR 20010108 18632729
K27ac_70CR 20009970 18287088
K27ac_78CR 20011916 17652472
K27ac_81CR 20011698 16457565
K27ac_96CR 20011149 15902411

Antibodies

Murine ChIP-seq:
AR: #ab108341, Abcam (lot #GR139151-3)
FOXA1: #ab23738, Abcam (lot #GR176970-1)
H3K27ac: #ab4729, Abcam (lot #GR167929-1)
RNA Pol II: #sc899, Santa Cruz Biotechnology (lot #H1114)
LuCaP PDXs ChIP-seq:
AR: N-20; #sc-816, Santa Cruz Biotechnology
FOXA1: #ab23738, Abcam
H3K27ac: #C15410196, Diagenode

Peak calling parameters

Peak calling is used the Model-Based Analysis of ChIP-seq 2 (MACS v2.1.1.20160309), with a q-value (FDR) threshold of 0.01.

Data quality

We evaluated multiple quality control criteria based on alignment information and peak quality: (i) sequence quality score; (ii) uniquely mappable reads (reads that can only map to one location in the genome); (iii) uniquely mappable locations (locations that can only be mapped by at least one read); (iv) peak overlap with Velcro regions, a comprehensive set of locations – also called consensus signal artifact regions – in the genome that have anomalous, unstructured high signal or read counts in next-generation sequencing experiments independent of cell line and of type of experiment; (v) number of total peaks (the minimum required was 1,000); (vi) high-confidence peaks (the number of peaks that are tenfold enriched over background); (vii) percentage overlap with known DHS sites derived from the ENCODE Project (the minimum required to meet the threshold was 80%); and (viii) peak conservation (a measure of sequence similarity across species based on the hypothesis that conserved sequences are more likely to be functional).

Software

ChiLin(http://cistrome.org/chilin/) for QC; BWA(http://bio-bwa.sourceforge.net/) for mapping; MACS2(http://liulab.dfci.harvard.edu/MACS/) for peak calling.
